# Concern about chemotherapy in oncological patients first referred to this treatment predicts negative emotions

**DOI:** 10.1192/j.eurpsy.2021.1158

**Published:** 2021-08-13

**Authors:** V. Sadovnichaja, E. Ledin, M. Kovyazina, S. Kirsanova

**Affiliations:** 1 Clinical Psychology, Moscow State University, Moscow, Russian Federation; 2 Oncology, MedSi, Moscow, Russian Federation

**Keywords:** chemotherapy, treatment representation, emotions

## Abstract

**Introduction:**

Treatment representation is an important factor of motivation and well-being during treatment (Horne, 2002).

**Objectives:**

The aim was to reveal the relationship between treatment representation and well-being in oncological patients first referred to chemotherapy.

**Methods:**

40 oncological patients (10 males, 20-72 years old, mean age 50.49±13.75 years old, localizations included gastrointestinal tract and genitourinary system) first referred to chemotherapy filled Satisfaction with Life Scale (Diener et al., 1985), Scale for Positive and Negative Experiences (Diener et al., 2009), Hospital Anxiety and Depression Scale (Zigmond, Snaith, 1983) and Beliefs about Medication Questionnaire (Horne, 2002) that was slightly modified for the situation of chemotherapy. Disturbance of functioning was assessed in the interview as an opportunity to cope with job, home responsibilities or self-care (1-5-point Likert scale).

**Results:**

Cronbach’s alphas for Necessity and Concern scales were .69 and .76. Despite high appraisals of necessity of chemotherapy (mean 4.24±.53 on 1-5 Likert scale), concern about it was rather high (2.83±.82). Hierarchical regression analyses revealed that, after adjusting for disturbances in social functioning, concern about chemotherapy (but not its subjective necessity) predicted more severe negative emotions (β=.32, p<.05, ΔR^2^=10.0%). After control for general level of anxiety and depression, this relationship became weaker but marginally significant (β=.32, p<.10, ΔR^2^=8.4%).

**Conclusions:**

Concern about chemotherapy in patients first referred to this treatment could be important predictor of well-being demanding for interventions aimed at stabilization of emotional reaction to chemotherapy regardless belief in its necessity.

